# Silicon nanoparticles in sustainable agriculture: synthesis, absorption, and plant stress alleviation

**DOI:** 10.3389/fpls.2024.1393458

**Published:** 2024-03-28

**Authors:** Guochao Yan, Qingying Huang, Shuaijing Zhao, Yunmin Xu, Yong He, Miroslav Nikolic, Nina Nikolic, Yongchao Liang, Zhujun Zhu

**Affiliations:** ^1^ College of Horticulture Science, Zhejiang Agriculture and Forestry University, Hangzhou, China; ^2^ Key Laboratory of Quality and Safety Control for Subtropical Fruit and Vegetable of Ministry of Agriculture and Rural Affairs, Zhejiang Agriculture and Forestry University, Hangzhou, China; ^3^ Collaborative Innovation Center for Efficient and Green Production of Agriculture in Mountainous Areas of Zhejiang Province, Zhejiang Agriculture and Forestry University, Hangzhou, China; ^4^ Institute for Multidisciplinary Research, University of Belgrade, Belgrade, Serbia; ^5^ Ministry of Education Key Laboratory of Environment Remediation and Ecological Health, College of Environmental and Resource Sciences, Zhejiang University, Hangzhou, China

**Keywords:** silicon nanoparticles (SiNPs), synthesis, uptake and translocation, biotic and abiotic stress, plant growth stimulator, nanocarrier, sustainable agriculture

## Abstract

Silicon (Si) is a widely recognized beneficial element in plants. With the emergence of nanotechnology in agriculture, silicon nanoparticles (SiNPs) demonstrate promising applicability in sustainable agriculture. Particularly, the application of SiNPs has proven to be a high-efficiency and cost-effective strategy for protecting plant against various biotic and abiotic stresses such as insect pests, pathogen diseases, metal stress, drought stress, and salt stress. To date, rapid progress has been made in unveiling the multiple functions and related mechanisms of SiNPs in promoting the sustainability of agricultural production in the recent decade, while a comprehensive summary is still lacking. Here, the review provides an up-to-date overview of the synthesis, uptake and translocation, and application of SiNPs in alleviating stresses aiming for the reasonable usage of SiNPs in nano-enabled agriculture. The major points are listed as following: (1) SiNPs can be synthesized by using physical, chemical, and biological (green synthesis) approaches, while green synthesis using agricultural wastes as raw materials is more suitable for large-scale production and recycling agriculture. (2) The uptake and translocation of SiNPs in plants differs significantly from that of Si, which is determined by plant factors and the properties of SiNPs. (3) Under stressful conditions, SiNPs can regulate plant stress acclimation at morphological, physiological, and molecular levels as growth stimulator; as well as deliver pesticides and plant growth regulating chemicals as nanocarrier, thereby enhancing plant growth and yield. (4) Several key issues deserve further investigation including effective approaches of SiNPs synthesis and modification, molecular basis of SiNPs-induced plant stress resistance, and systematic effects of SiNPs on agricultural ecosystem.

## 1 Introduction

Silicon (Si) is a typical metalloid and the second most abundant element in the earth’s crust (Epstein, 1994). Due to its growth promotion effects in plants, particularly for those grown under stressful conditions, Si is widely recognized as a plant beneficial element (Epstein, 1999; Liang et al., 2015). In recent years, with the progressive integration of agriculture and nanotechnology, the application of nanoparticles (NPs) is shown to be an effective agronomic approach in crop production to address the escalating global food demand (Agathokleous et al., 2020; Wang et al., 2020; Sharma et al., 2023). Among the various NPs, silicon nanoparticles (SiNPs) demonstrate impressive advantages and applicability in nano-enabled agriculture for promoting plant stress resistance and ensuring stable crop yield (Rastogi et al., 2019; Dhakate et al., 2022).

Technically, SiNPs refer to fabricated Si particles at nanoscale, with the dimensions ranging from 1 to 100 nm. Based on the structures of SiNPs, they can be categorized into various types such as spheric, hollow, shaped (e.g., rod, cube), and porous (Mathur and Roy, 2020). SiNPs exhibit significant advantages over bulk Si sources including high surface/volume ratio, distinct charge properties and improved plant bioavailability (Jeelani et al., 2020). The characteristics and structures of SiNPs are commonly determined by the synthesizing process, which can be classified into physical, chemical, and biological synthesis (green synthesis), depending on the driving force (Naidu et al., 2023). The green synthesis of SiNPs with agricultural wastes as raw materials is gaining growing attention owing to its significant applicability in recycling and sustainable agricultural production (Mahawar et al., 2023). In plants subject to foliar or root application of SiNPs, due to the size effect and charge property, SiNPs can directly penetrate plant barriers such as epidermis, cell wall and plasma membrane, subsequently being accumulated and translocated in plants (Jeelani et al., 2020; Wang et al., 2022a). In addition to the direct penetration, plants also utilize SiNPs in the form of silicic acid after dissolution under the facilitation of Si channels and transporters (e.g., Lsi1, Lsi2, Lsi3 and Lsi6) (Ma and Yamaji, 2015; Mandlik et al., 2020).

**Figure 1:**
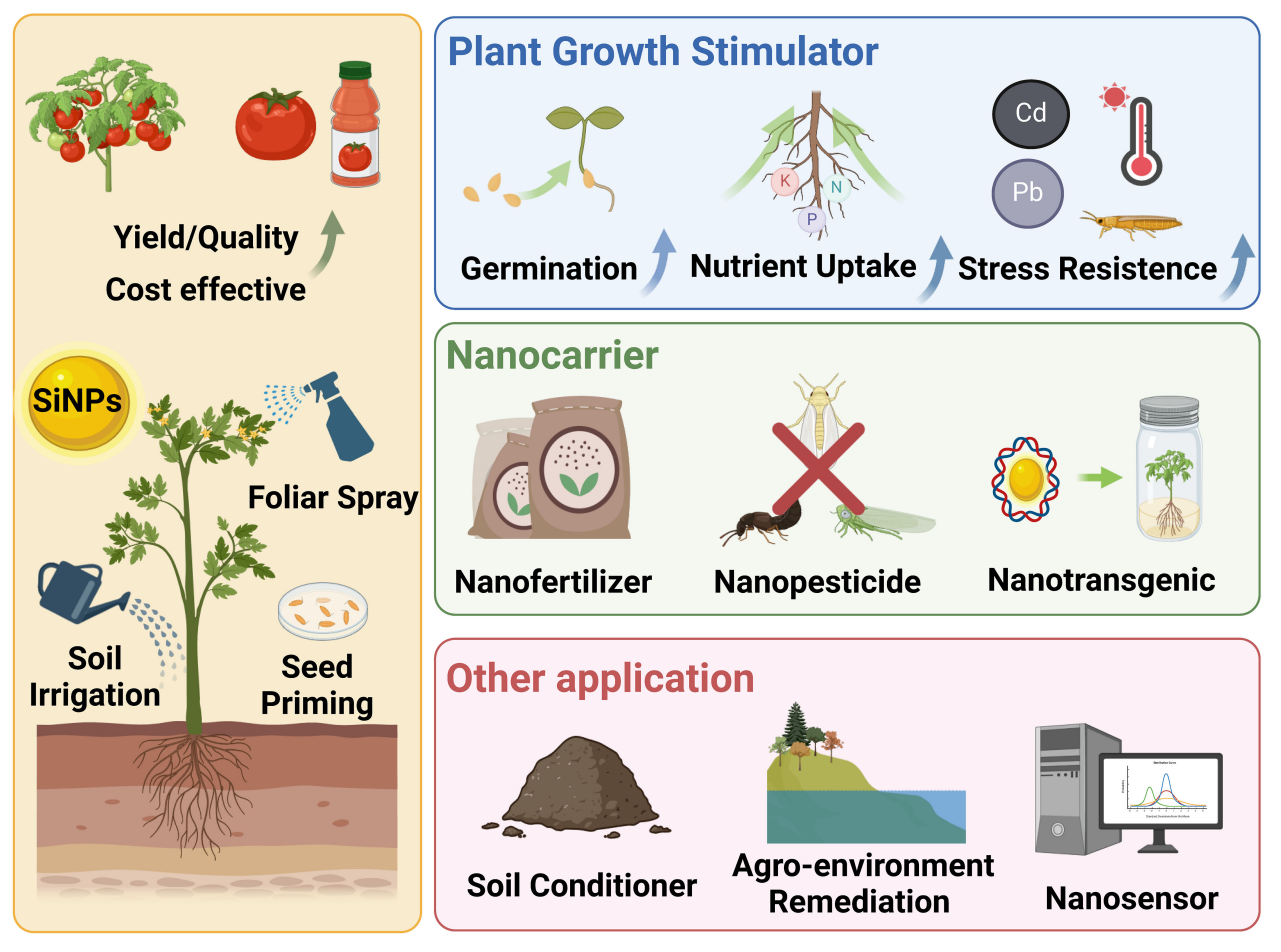
The application of SiNPs in nano-enabled agriculture.

The unique features and advantages of SiNPs including nanoscale sizes, nutritional effects, surface properties and porous nature, endowing them versatile functions in nano-enabled agriculture such as plant growth stimulator, nanocarrier, and soil conditioner (**Figure 1**; Ji et al., 2018; Rastogi et al., 2019; Mahawar et al., 2023). To date, numerous laboratory and field studies affirm that SiNPs, as plant growth stimulator, can enhance plant resistance to various biotic (e.g., insect pest, pathogen disease) and abiotic (e.g., metal stress, drought stress, salt stress) stress, thereby promoting plant growth, yield and quality (Bansal et al., 2022; Verma et al., 2022; Wang et al., 2022a). In addition, the porous nature of SiNPs makes them ideal carriers for delivering chemicals (e.g., fertilizer, pesticide, plant growth regulator) and bioactive molecules (e.g., DNA, protein) in agricultural production and plant biotechnology (Mathur and Roy, 2020; Zhang et al., 2023). Furthermore, SiNPs can also be applied for the improvement of soil properties, detection and monitoring of certain biochemical parameters relevant for agronomic production, and remediation of agricultural contamination (Giraldo et al., 2014; Kannan and Sujatha, 2022).

Overall, SiNPs show significant applicability to sustainable agricultural production, while rapid progress has been made to unveil the interaction between SiNPs and crops, especially for those under stressful conditions in the recent decade. This review provides an updated summary of the synthesis and application of SiNPs in agriculture, their uptake and accumulation in plants under foliar and root application, and the multiple roles and underlying mechanisms of SiNPs in protecting plants against biotic and abiotic stresses. In addition, several key issues related to the current limitations and future perspectives of SiNPs research and application in nano-enabled agriculture are highlighted.

## 2 SiNPs synthesis

**Figure 2:**
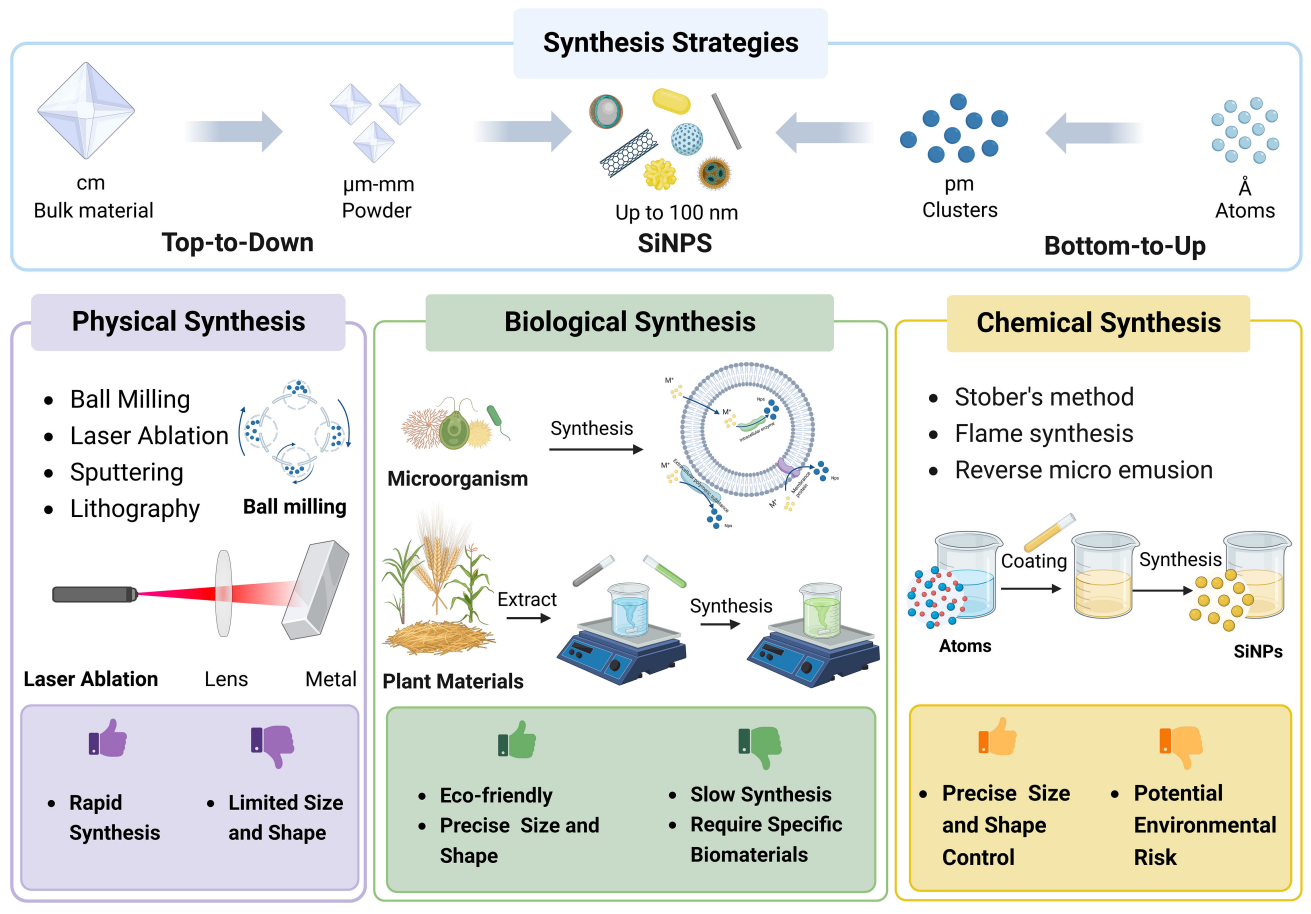
The strategies and approaches of SiNPs synthesis.

The unique characteristics and attributes of SiNPs are predominantly dependent on precursors and methods employed in their synthesis processes (Jeelani et al., 2020). Presently, two strategies called top-to-down strategy and bottom-to-top strategy are mostly applied in the synthesis of NPs (**Figure 2**; Vetchinkina et al., 2019; Salami et al., 2022). The top-to-down strategy indicates the breakdown of larger materials into smaller particles using methods such as physical milling and chemical decomposition. Conversely, the bottom-to-up strategy involves the assemble of atomic or molecular precursors into complex nanostructures based on natural physical principles or external forces (Rahman and Padavettan, 2012; Tang et al., 2012).

According to the driven forces and precursors involved, the synthesis processes of NPs can be classified into physical, chemical, and biological (green synthesis) approaches (**Figure 2**; Usman et al., 2020; Zhao et al., 2020). Typically, physical and chemical synthesis are conducted using silicate precursors such as tetraethyl orthosilicate (TEOS) and tetramethyl orthosilicate (TMOS), while green synthesis usually utilizes plants and microorganisms (Tang et al., 2012). Physical methods include ball milling, ultrasonic peening, and laser ablation, while chemical methods encompass vapor condensation, microemulsion, co-precipitation, and sol-gel method (Debnath et al., 2012; Croissant et al., 2020). In most biogenic synthesis of SiNPs using plant materials, the process involves two steps including the extraction of silicate and the formation of SiNPs through the sol-gel method using inorganic salt neutralization with hydrochloric acid (Seroka et al., 2022).

Although the physical methods are relatively simple and direct with quick synthesis processes, they may be limited by prosomal material and usually result in nanoparticles with uncontrollable sizes. On the other hand, the chemical methods offer better control of particle size and functional manipulation, however, the usage of chemicals with potential toxicity could raise environmental risks (**Figure 2**). In contrast, the green synthesis using agricultural wastes such as rice straw and husk (Wang et al., 2012a; Gu et al., 2015; Bose et al., 2018), maize stalk (Adebisi et al., 2020; Piela et al., 2020), sugarcane bagasse (Alves et al., 2017), and coconut shell (Marousek et al., 2022) shows significant advantages over physical and chemical synthesis approaches. Especially, green synthesis demonstrates a significant potential for the large-scale production of SiNPs in recycling and sustainable agricultural production (Mahawar et al., 2023).

## 3 SiNPs absorption and translocation in plant

### 3.1 Si uptake and translocation

In the nature, Si mainly exists in the forms of SiO_2_ and silicates and silicic acid. As the soluble and sole form of Si that can be absorbed by plants root, mono-silicic acid would form silicates when the environmental pH > 9 (Epstein, 1994). In arable soils, the concentration of mono-silicic acid ranges from 0.1 to 0.6 mM (lower than its saturation solubility, about 2 mM), which is mainly determined by parent materials, development levels, and physical and chemical properties of soil (Liang et al., 2015; Yan et al., 2018). After being absorbed by root, most of Si (more than 90%) is loaded in xylem and transported from root to shoot in the form of silicic acid and then unloaded to parenchyma cells. In plants’ shoot, along with water loss driven by plant transpiration, silicic acid is polymerized to silica gel (SiO_2_·nH_2_O) and forms cuticle-silica double layers (Mandlik et al., 2020). In plants, the accumulation of Si in the above-ground part differs greatly, ranging from 0.1 to 10.0% on dry weight basis. Accordingly, plants have been artificially clarified into three categories including high accumulator (1.5-10%), moderate accumulator (0.2-1.5%), and low accumulator (lower than 0.2%, also called extruder). On the molecular level, the difference of existence, function and activity of Si transport proteins are shown to be responsible for the distinct abilities of Si accumulation among different plant species (Mitani-Ueno and Ma, 2021).

To the date, a molecular model of Si transport in higher plants responsible for Si uptake, translocation, distribution and accumulation has been sketchily established in rice, a typical Si accumulator and model plant in Si researches following the identification of Si channels and transporters including Lsi1, Lsi2, Lsi3 and Lsi6 based on mutant selection and forward genetics (Ma and Yamaji, 2015; Mitani-Ueno and Ma, 2021). OsLsi1 was the very first protein responsible for Si uptake in higher plants using a rice mutant (low silicon 1, *lsi1*), which belongs to the noduline-26 major intrinsic protein (NIP) family and act as an SI influx channel (Ma et al., 2002, Ma et al., 2006). Then, a Si efflux transporter (OsLsi2) was identified, which belongs to putative anion-channel transporter family (Ma et al., 2007). OsLsi1 and OsLsi2 are localized to the plasma membrane of exodermis and endodermis cells, while both proteins show polar localization (OsLsi1 at distal side and OsLsi2 at proximal side), and the cooperation of OsLsi1 and OsLsi2 facilitate Si uptake in rice root. OsLsi6, a homolog of OsLsi1, is localized at the adaxial side of xylem parenchyma cells and responsible for the unloading process of Si from xylem to arial parts (Yamaji et al., 2008; Yamaji and Ma, 2009). Beside the three proteins mentioned above, OsLsi3, a homolog of OsLsi2, takes the charge of controlling the distribution between panicles and flag leaves in cooperation with OsLsi2 and OsLsi6 in rice node (Yamaji et al., 2015).

### 3.2 SiNPs absorption and translocation

The absorption and translocation of SiNPs significantly affects their efficacy as plant growth stimulator and nanocarrier in plants and agricultural production (Lombi et al., 2019; Usman et al., 2020). However, in contrast with the relatively well-understood mechanisms of Si transport, the uptake and translocation of SiNPs in plants, especially at the subcellular and molecular level, remains unclear. Moreover, the research on the SiNPs uptake and translocation in plants also lags those on metal NPs such as silver nanoparticles (AgNPs), gold nanoparticles (AuNPs), cerium nanoparticles (CeNPs), and iron nanoparticles (FeNPs). The issue of phytotoxicity of metal NPs has attracted much research attention; on the contrary, no convincing evidence has confirmed the toxicity of SiNPs (Miralles et al., 2012; Sharma et al., 2015; Ruttkay-Nedecky et al., 2017).

**Figure 3:**
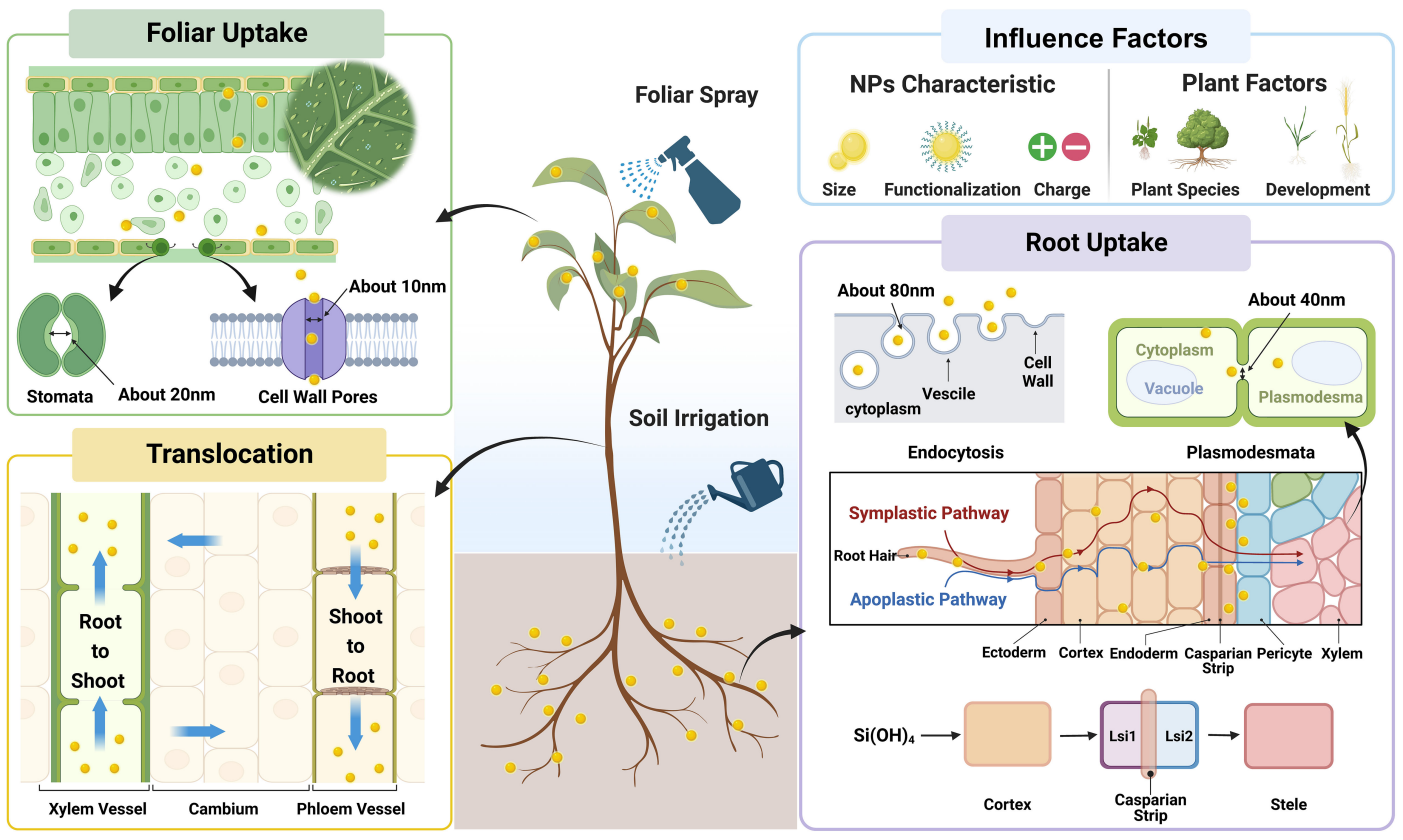
The uptake and translocation of SiNPs in plants.

In general, NPs can directly enter plant shoot and root tissues due to their nanoscale sizes when supplied with foliar spray and root application, respectively (**Figure 3**; Lv et al., 2019; Rastogi et al., 2019). When supplied via foliar spray, NPs are usually absorbed through cuticle and/or stomata (Yeats and Rose, 2013). It has been estimated that the maximum size of NPs that can pass through cuticle is approximately 5 nm (Eichert and Goldbach, 2008), while the equivalent pore size of stomata is approximately 20 - 500 nm (Eichert et al., 2008). Given the fact that most of NPs used in agricultural practices are larger than 5 nm, the stomatal pathway could play a dominant role in NPs uptake under foliar application (**Figure 3**). In the case of root application, the uptake of NPs usually occurs in the immature parts of root such as root tips, root hairs, and lateral root junctions, where the physical barriers (e.g., Casparian strip, suberin lamella) are underdeveloped (Wang et al., 2022a). Additionally, SiNPs may be transformed into silicic acid in growth substance driven by geochemical, microbial, and plant biological factors, which can successively be taken up by plant roots through Si transport proteins (Ma and Yamaji, 2015; Mandlik et al., 2020). Once taken up by plant shoot or root, the shoot-to-root and root-to-shoot translocation of NPs subsequently occurs in phloem and xylem, respectively (**Figure 3**; Wang et al., 2012b; Ma et al., 2017). Moreover, it should be noted that most of the plant species are low Si accumulators with relatively poor ability in Si uptake and accumulation, and could benefit from Si application at relatively lower levels (Liang et al., 2015; Coskun et al., 2019). In contrast, due to different absorption and transport mechanisms with Si, the limitations would not exist under the application of SiNPs, since most of SiNPs are absorbed independently to Si transport proteins. Therefore, as a novel Si source, SiNPs show remarkable advantages and applicability, and may play more significant role in future, while the further research comparing SiNPs and bulk Si materials are still needed.

### 3.3 Factors influencing SiNPs absorption

It can be concluded from previous literatures that, in essence, the uptake and translocation of NPs in plants is the process of NPs passing through plant biological barriers such as cuticle, stomata, cell wall and vascular vessels. Therefore, it is not surprising that both NPs properties (e.g., size and charge property) and plant factors (plant species, growth and development stage) would influence the uptake and distribution of NPs in plants (**Figure 3**; Ma et al., 2010; Tripathi et al., 2017). The size preference of plants in NPs uptake and translocation has been documented in different plant species (e.g., wheat, cucumber, and tobacco), that NPs with smaller size are more easily absorbed by plants (Judy et al., 2012; Hong et al., 2014). However, unlike that of size properties, the effects of charge properties of NPs on their absorption and translocation are more complicated, which also differs between root and foliar applications. Under root application, the positively charged NPs adhere to root surface more tightly, while those with negative charge are more efficiently translocated from root to shoot (Zhu et al., 2012; Avellan et al., 2017; Spielman-Sun et al., 2017). However, under foliar application, Hu et al. (2020) reported that NPs with positive charge showed the highest delivery efficiency into stomata and apoplastic space. Moreover, the uptake and translocation of NPs is also influenced by plant factors since several key parameters differ with plant species and development stages including (1) the contact area between plant and NPs based on plant morphological traits; (2) the amount of immature roots and leaves which are the major entrance of NPs into plants; (3) the equivalent pore sizes of physiological barriers such as cell wall, plasma membrane, cuticle, stomata, and vascular vessels) (Tripathi et al., 2017; Lv et al., 2019; Dhakate et al., 2022). Overall, current literature is largely based on the determination and/or observation of NPs in plants, while the mechanisms of NPs uptake and translocation, especially at cellular and molecular levels, remain unclear and deserve further investigation.

## 4 SiNPs and biotic stress

### 4.1 Direct effects of SiNPs on pathogens and pests

**Figure 4:**
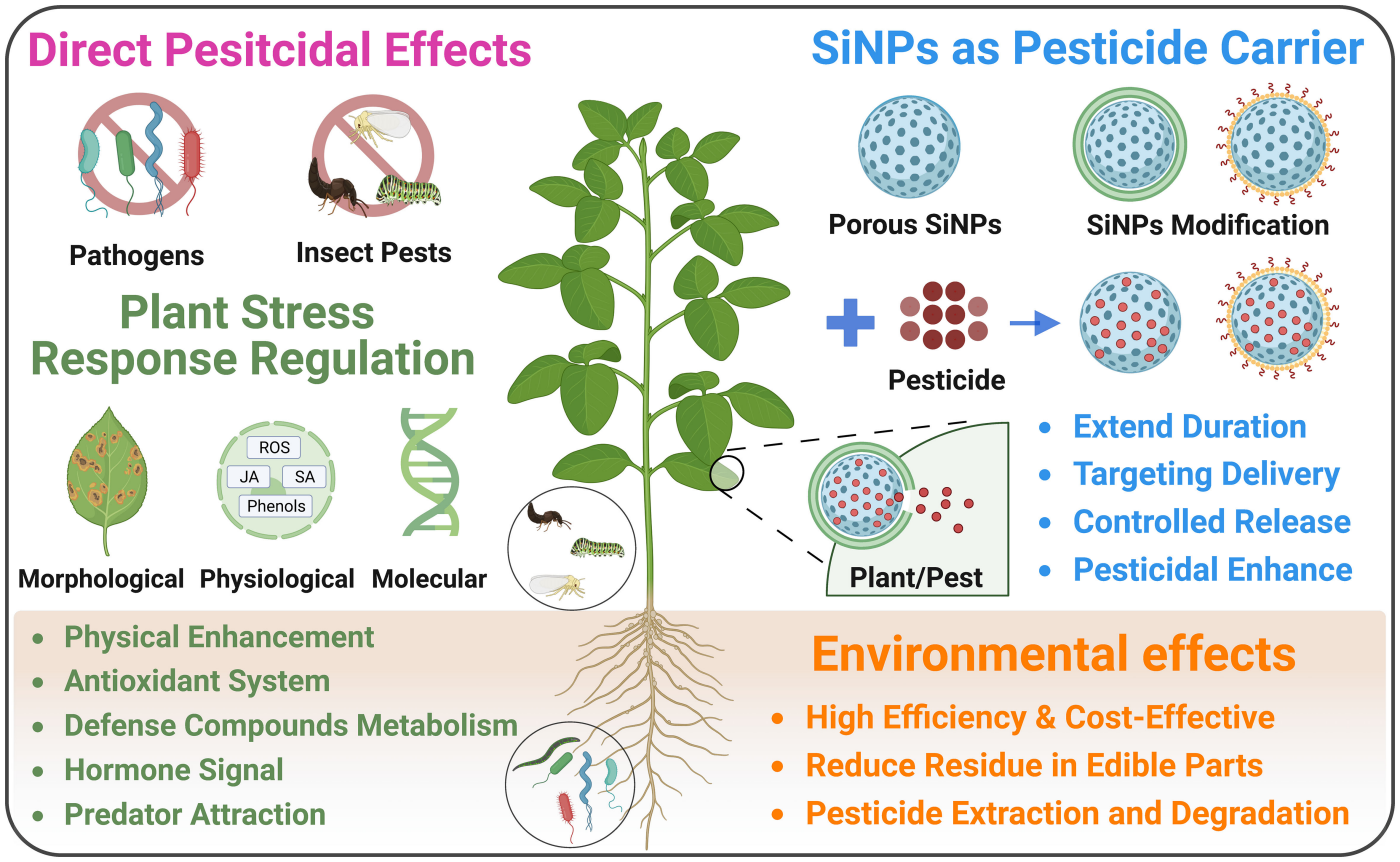
Effects of SiNPs in agricultural production under biotic stress.

As an eco-friendly biocide, SiNPs restrain the growth and aggressiveness of pathogens and insect pests, thereby protecting plant against the attack of bacteria, fungi, and pests in agricultural production (**Figure 4**; Selvarajan et al., 2020; Goswami et al., 2022). For instance, it was shown that SiNPs induced significant antifungal effects against *Rhizoctonia solani* and *Alternaria solani* in the incubation experiments (Abdelrhim et al., 2021; Albalawi et al., 2022). In addition, Si/AgNPs (complex NPs of Si and Ag) also showed remarkable fungicidal and bactericidal effects against various plant pathogens such as *Botrytis cinerea*, *Rhizoctonia solani*, *Pseudomonas syringae* and *Xanthomonas campestris* (Park et al., 2006; Baka and El-Zahed, 2022). Furthermore, Khan et al. (2022) reported that both SiNPs and titanium nanoparticles (TiNPs) inhibited the growth of *Phomopsis vexans* and *Ralstonia solanacearum*, while SiNPs induced a more prominent recuction of pathogen growth than TiNPs. As for insect pests, in a surface contact and feeding experiment, Ayoub et al. (2017) found that SiNPs induced remarkable pesticidal effects in leafworm (*Spodoptera littoralis*), which was also influenced by their size and surface characteristics. Notably, SiNPs demonstrate unique advantage over traditional pesticides in that their pesticidal effects are based on the physical effects, implying pathogens and insect pests are unlikely to become resistant to SiNPs at physiological level through evolution.

### 4.2 SiNPs enhance plant resistance to biotic stress

In addition to the direct pesticidal effects, SiNPs treatment with seed priming and foliar application can enhance seed germination, and plant growth and yield under different biotic stresses (**Figure 4**; Naidu et al., 2023; Saw et al., 2023). Seed priming with SiNPs is an effective agronomic approach in enhancing seed germination and plant growth under the infection of pathogens. For example, SiNPs application via seed priming significantly promoted seed germination and seedling growth in wheat infected by *Rhizoctonia solani* (Abdelrhim et al., 2021) and watermelon infected by *Fusarium oxysporum* (Buchman et al., 2019). Besides, foliar application of SiNPs is more commonly used in agricultural practices in protecting plants against pathogens and pests. It has been reported that SiNPs foliar spray alleviated pathogen-induced growth inhibition and disease symptoms in plants under the infection of various pathogens and insect pests such as *Ralstonia solanacearum* (Khan et al., 2022; Wang et al., 2022b), *Alternaria solani* (Albalawi et al., 2022), *Plasmopara viticola* (Rashad et al., 2021), *Fusarium oxysporum* (Kang et al., 2021), *Mythimna separata* (Wang et al., 2021), *Aphis craccivora and Spodoptera littoralis* (Thabet et al., 2021).

The SiNPs-induced broad-spectrum biotic stress resistance in plants is largely based on the regulation of SiNPs on plant defense system at physiological and molecular levels such as enhancement of defense compounds metabolism, modulation of antioxidant system, and regulation of plant hormone signals (**Figure 4**). For instance, Suriyaprabha et al. (2014) reported that SiNPs was more effective than bulk Si in enhancing the resistance of maize to fungal pathogens including *Fusarium oxysporum* and *Aspergillus niger* through regulating the metabolism of phenolic compounds. As for insect pests, Wang et al. (2021) found that SiNPs enhanced the metabolism of chemical defense compounds such as chlorogenic acid and total phenolics, and protected maize against oriental armyworm. In a field test, SiNPs treatment reduced the population of three typical pests in faba bean and soybean via enhancing the attraction of the predators of pests, which could be due to the regulation of the metabolism of volatile compounds (Thabet et al., 2021). Furthermore, it has been documented that SiNPs regulated the activity of antioxidant enzyme and non-enzymatic antioxidant, thereby ensuring the homeostasis of reactive oxygen species (ROS) in eggplant under the infection by *Alternaria solani* (Albalawi et al., 2022) and wheat under the infection oby *Rhizoctonia solani* (Abdelrhim et al., 2021). Considering the dual roles of ROS in plants under stress including signal molecule and toxic radicals, the regulation of SiNPs on ROS balance could participate in both pathogen recognition and oxidative damage alleviation. Besides ROS, SiNPs can regulate the plant hormones signal pathways such as salicic acid (SA), jasmonic acid (JA) and ethylene, which play pivotal roles in biotic stress response and accilimation. It has been documented that SiNPs foliar spary enhanced SA metabolism via regulating the expression of related genes in tomato grown under the infection of *Ralstonia solanacearum* (Wang et al., 2022b). Moreover, Rashad et al. (2021) reported that SiNPs promoted the resistance of grapevine to *Plasmopara viticola* by regulating jasmonate and ethylene signal pathway, and enhacing the expression of pathogen defense-related genes. Particularly, SiNPs can trigger SA signal via relasing silicic acid or clogging stomata, thereby inducing systemic acquired resistance (SAR, a typical plant immune response under pathogen infection) and enhacing the resistance of *Arabidopsis* against *Pseudomonas syringae* (El-Shetehy et al., 2021).

### 4.3 SiNPs as pesticide carrier

Although both bulk Si and SiNPs can alleviate biotic stress in plants acting as plant growth stimulator, SiNPs demonstrate distinct usage in plant protection. They can be used as vehicle in delivering pesticides in virtue of their porous nature, while the pesticide loaded SiNPs exhibit kinds of advantages over direct pesticide application (**Figure 4**). Generally, SiNPs can be applied to deliver pesticides directly or after certain modification in agricultural practices. The uptake efficiency and durability of pesticides in plants would be enhanced when loaded into SiNPs, therefore improving their pesticidal effects. For example, Bilal et al. (2020) reported that indoxacarb-loaded SiNPs exhibited better insecticidal activity than commercial indoxacarb in inhibiting *Plutella xylostella* when applied at the same dose. The usage of pectin coated SiNPs as carrier promoted the uptake, translocation, duration, and antifungal activity of prochloraz in rice (Abdelrahman et al., 2021). It has also been suggested that α-cyclodextrin anchored SiNPs enhanced the light- and thermal- shielding ability of avermectin after being loaded, thereby prolonging the duration of avermectin in controlling *Plutella xylostellla* (Kaziem et al., 2018). Furthermore, the surface modification of SiNPs using copper (Cu) or carboxymethyl chitosan enhanced their translocation in plants and extended the release period of azoxystrobin (Xu et al., 2018, Xu et al., 2020).

On the other hand, the application of SiNPs as a vehicle can improve the targeting precision and foster a controlled release of pesticide in plants or insect pests after specific modifications (**Figure 4**). For instance, Chen et al. (2016) fabricated a SiNPs-based chlorpyrifos release system with salicylaldehyde or Cu modification, which showed significant pH sensitivity and sustained pesticide release. Similarly, Gao et al. (2019) developed a pH-sensitive abamectin release system based on SiNPs after 3-(trimethoxysilyl)propyl methacrylate functionalization, which exhibited higher affinity for rice leaves, longer duration period of, and higher toxicity to the larvae of *Cnaphalocrocis medinalis* in contrast with commercial abamectin. In their further research, Gao et al. (2020) developed a temperature-responsive pesticide release formulation based on SiNPs using thermo-responsive copolymer, which showed stronger adhesion to rice leaves and long-term bioactivity of thiamethoxam. Moreover, Liang et al. (2020) modified SiNPs using functionalized starch with biodegradable disulfide-bridged structure, and then loaded avermectin into SiNPs. The results showed the modified SiNPs controlled the release of avermectin in response to glutathione and α-amylase, thereby enhancing the targeting pesticidal effects against *Plutella xylostella*. Analogously, the encapsulation of acetamiprid and decanethiol in SiNPs would also control the release of acetamiprid in response to glutathione and induce higher pesticidal effects in contrast with commercial acetamiprid (Ding et al., 2023). In the study of Bapat et al. (2020), the triethoxysilane-functionalized SiNPs was used as vehicle for delivering trypsin inhibitor, which would release pesticide once being transported to the gut of *Helicoverpa armigera*, thereby effectively inhibiting the activity of gut proteinase and the growth of this pests.

Notably, the excessive application of synthetic pesticide in agricultural practice threatens food safety and the sustainability of agricultural production. Under pesticide contamination in agriculture, SiNPs can decrease pesticide residues in edible parts, and be used for pesticide extraction and degradation in environmental remediation (**Figure 4**; Bapat et al., 2016). For instance, it has been reported that the usage of SiNPs as carrier in delivering prochloraz and spirotetramat decreased the final pesticide residue and related metabolites in the edible parts of cucumber (Zhao et al., 2018a, Zhao et al., 2018b). In the case of pesticide extraction and degradation, Korrani et al. (2016) reported that SiNPs effectively extracted three organic phosphorus pesticides including dicrotophos, chlorpyrifos and diazinon from water samples, which could be due to its mesoporous nature and high surface area. Amani et al. (2018) reported that SiNPs can be used for the removal of diazinon in solution, while the modification of SiNPs with propyl methacrylate enhanced the removal efficiency. Moreover, Yang et al. (2016) used SiNPs to immobilize laccase for degradation of 2,4-dichlorophenol, and the results indicated that the application of SiNPs enhanced the efficiency of degradation and reusability of laccase. However, it should be noted that most of the previous research using SiNPs for pesticide extraction and degradation were conducted in aqueous solution, while the potential application of SiNPs in agricultural soil for environmental remediation deserves further investigation.

## 5 SiNPs and abiotic stress

### 5.1 Metal stress

**Figure 5:**
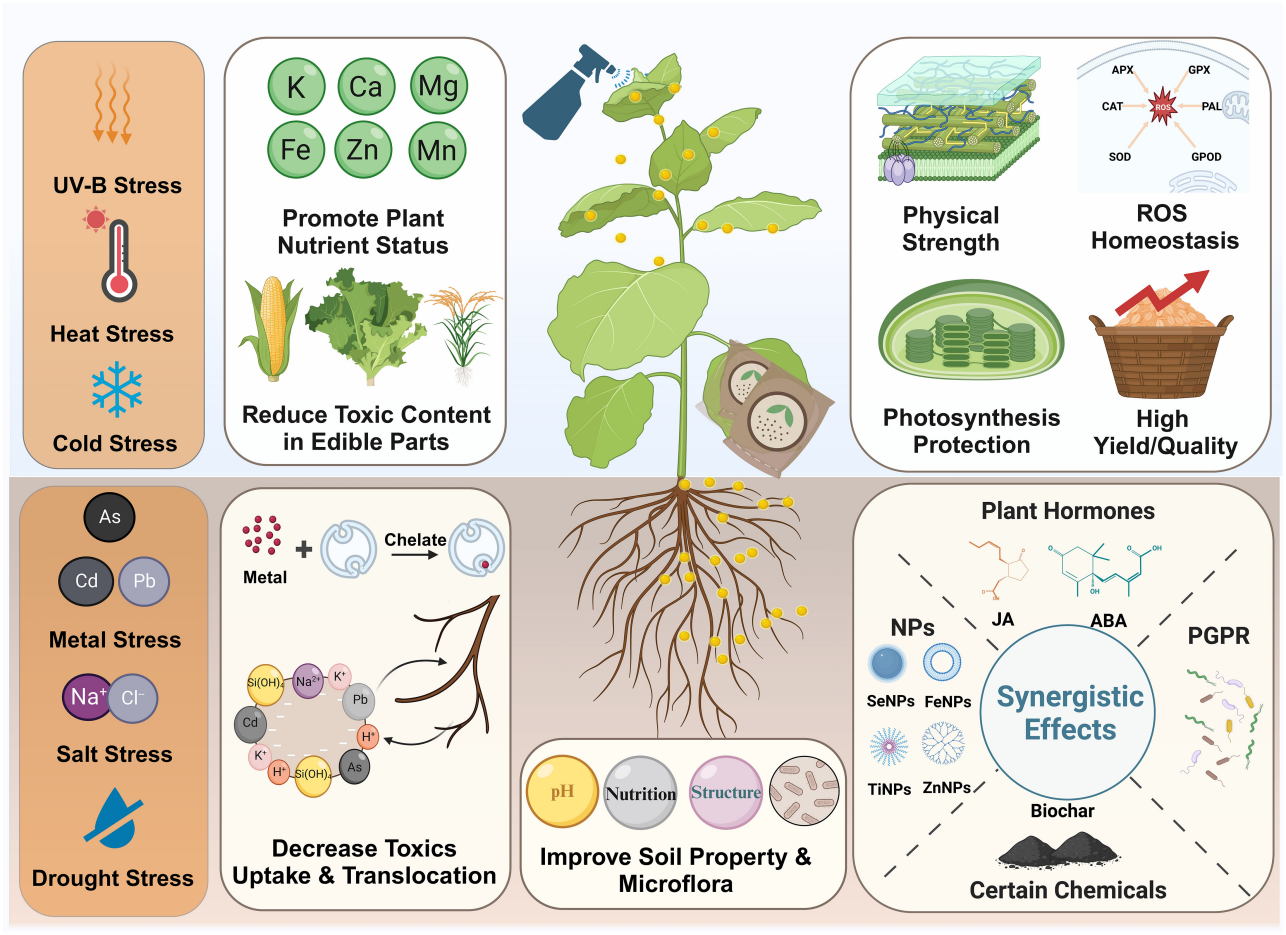
Effects of SiNPs in agricultural production under abiotic stress.

Under the growing influence of human activities (e.g., mining, chemical fertilizer application and sewage irrigation) on agriculture, metal contamination has become one of the major threats in sustainable agricultural production and food safety (Clemens and Ma, 2016; Rai et al., 2019). Metal stresses induce oxidative damages, nutritional imbalance, photosynthesis system destruction, and plant growth inhibition (Clemens et al., 2002). As a promising tool in protecting plant against metal stress, SiNPs are proven to effectively ameliorate various metal toxicity such as cadmium (Cd) (Riaz et al., 2022a; Zhao et al., 2023), arsenic (As) (Gonzalez-Moscoso et al., 2022; Yang et al., 2022), mercury (Hg) (Li et al., 2020), lead (Pb) (Hussain et al., 2020), Cu (Riaz et al., 2022b), aluminum (Al) (de Sousa et al., 2019), and chromium (Cr) (Tripathi et al., 2015). Multiple mechanisms are involved behind the SiNPs-induced broad-spectrum metal stress tolerance (**Figure 5**).

SiNPs can decrease the accumulation of metals in plants, especially in arial or edible parts, under metal contamination (Okeke et al., 2023; Yadav et al., 2023). For example, in wheat grown under Cd exposure, SiNPs application through seed priming (Hussain et al., 2019), foliar spray, and soil application (Ali et al., 2019) significantly promoted plant growth and decreased Cd accumulation in wheat grains. In addition, Yang et al. (2022) reported that SiNPs application increased As accumulation in rice shoot and husk but decreased As content in grain under As contamination, while Tripathi et al. (2015) found that SiNPs reduced the accumulation of Cr in both shoot and root in pea seedling growth under Cr stress. As for the underlying mechanisms, it has been documented that SiNPs treatment reduced Cd accumulation in rice shoot via enhancing polysaccharides metabolism and cell wall retention (Riaz et al., 2022a). Moreover, Yan et al. (2023) indicated that SiNPs were more effective than Si in reducing apoplastic flow of Cd uptake, thereby decreased Cd accumulation tomato shoot exposed to Cd. By using suspension rice cells, Cui et al. (2017; 2020) investigated the effects of SiNPs on Cd and As accumulation and related mechanisms, and found that SiNPs regulated the expression of genes responsible for Cd transport (*OsLCT1*, *OsNRAMP5* and *OsHMA3*) and chemical components of the cell wall, thereby reducing the accumulation of Cd and As in rice cells.

In addition to the reduction of toxic metal uptake and translocation, SiNPs can modulate the activity of antioxidant system and maintain mineral nutrients homeostasis, successively promoting plant growth and yield. Under Cd exposure, SiNPs application enhanced ROS scavenge and ameliorated oxidative injury in wheat and rapeseed via increasing the activity of antioxidant enzymes such as superoxide dismutase (SOD), catalase (CAT), ascorbate peroxidase(APX), and peroxidase (POD) (Adrees et al., 2022; Ahmed et al., 2023). Additionally, it has been shown that SiNPs alleviated Cd-induced oxidative damage in rapeseed by modulating the metabolism of antioxidants including ascorbate, glutathione, and proline (Zhao et al., 2023). The maintenance of mineral nutrient balance plays a key role in plant growth and development when grown under metal stresses, while it has been demonstrated that SiNPs was able to enhance mineral nutrition status including zinc (Zn), manganese (Mn), iron (Fe), magnesium (Mg), calcium (Ca), and potassium (K), resulting in promoted Cd stress resistance in barley (He et al., 2023). Similarly, in Phaseolus vulgaris under Cd stress, Koleva et al. (2022) reported that the application of SiNPs enhanced K uptake, polyamines biosynthesis and photosynthetic capacity, thereby promoting plant growth.

### 5.2 Salt stress

Salt stress is a major abiotic stress in agricultural production, affecting approximately 20% total arable land worldwide (Byrt and Munns, 2008). The alleviative effects of SiNPs on salt stress have been observed in various crops such as rice (Shalaby et al., 2021), wheat (Hajihashemi and Kazemi, 2022), maize (Rizwan et al., 2023), tomato (Sayed et al., 2022), cucumber (Alsaeedi et al., 2018), and potato (Gowayed et al., 2017). Under salt stress, the germination of plant seed is inhibited due to the water uptake limitation and ionic toxicity, while SiNPs priming can improve seed germination in cucumber (Alsaeedi et al., 2018), lentil (Alsaeedi et al., 2018), and maize (Naguib and Abdalla, 2019) which could be based on their regulation on K/Na ratio, ROS homeostasis and hormone metabolism in seeds. Besides seed germination, SiNPs can promote plant growth under salt stress through modulating Na/K homeostasis, antioxidant system, photosynthesis performance, and the expression of stress-response genes under salt stress (Etesami et al., 2021; Muhammad et al., 2022) (**Figure 5**).

In plant grown under saline condition, salt stress induces ion toxicity and osmotic constraint, thereby affecting plant growth, yield, and quality (Munns, 2005; Munns and Tester, 2008). The homeostasis of Na/K plays the dominant role in plant salt stress resistance, while it has been documented that SiNPs application regulated Na/K balance in rice and sweet orange via regulating the expression of Na/K transporter genes including *HKT*, *SOS*, and *NHX* (Mahmoud et al., 2022; Ijaz et al., 2023). Moreover, it was found that SiNPs eliminated the accumulation of MDA and H_2_O_2_ caused by salt stress, via enhancing the activity of antioxidant enzymes such as glutathione reductase (GR), APX, CAT, POD, SOD in squash (*Cucurbita pepo* L.) (Siddiqui et al., 2014) and pea (Ismail et al., 2022). In tomato grown under hydroponic condition, Haghighi and Pessarakli (2013) found that SiNPs can improve photosynthetic rate, mesophyll conductance, and photosynthetic water use efficiency, thereby promoting plant growth and salt stress resistance. In addition, Alam et al. (2022) reported that SiNPs promoted tomato growth, enhanced mineral nutrients accumulation (e.g., Mg, K, Fe, Mn, Zn) and photosynthesis performance, while foliar application was more effective in ameliorating salt stress in tomato than root dipping.

### 5.3 Drought stress

Drought stress is another major abiotic stress adversely affecting agricultural production. The alleviation effects of SiNPs on drought stress have been repeatedly documented, with diverse mechanisms involved (**Figure 5**). For instance, SiNPs promoted leaf area, chlorophyll content, and nitrogen assimilation, thereby enhancing plant growth and fruit yield in cucumber (Alsaeedi et al., 2019). Similarly, Aqaei et al. (2020) reported that foliar application of SiNPs on maize ameliorated drought stress-induced mineral nutrients imbalance and enhanced corn weight. In a field study, Namjoyan et al. (2020) indicated that SiNPs treatment at 1 mM improved shoot water status, enhanced photosynthesis rate and glycine betaine metabolism, and regulated the activities of antioxidant enzymes including SOD, CAT and GPX in sugar beet.

Moreover, the comparative effects of Si and SiNPs on drought stress tolerance in plants have been investigated in previous studies. Rai-Kalal et al. (2021) demonstrated that SiNPs priming was more efficient than that of bulk Si in promoting seed germination, seedling growth, chlorophyll fluorescence index in wheat under drought stress. In strawberry, Zahedi et al. (2023) reported that both Si and SiNPs improved drought stress tolerance through regulating photosynthesis performance and modulating the metabolism of carbon and plant hormone, while the regulatory effects differed between Si and SiNPs. Additionally, Ghorbanpour et al. (2020) compared the roles of Si and SiNPs in drought stress recovery in barley and found that SiNPs application more efficiently promoted barley growth, modulated antioxidant enzyme activity, and regulated the metabolism of osmolytes than bulk Si application.

### 5.4 Synergistic effects of SiNPs in alleviating abiotic stress

In agricultural practice, SiNPs can also be applied along with other plant growth regulators such as plant growth promoting rhizobacteria (PGPR), plant hormones, other NPs, and plant growth stimulating chemicals, which is more effective in enhancing plant abiotic stress resistance (**Figure 5**). As for PGPR, Eltahawy et al. (2022) indicated that the integrated application of heavy metal-resistant bacteria and SiNPs more efficiently regulated antioxidant system and promoted spinach growth under metal contamination than individual application of SiNPs. In wheat grown under semi-arid condition, the combined treatment of SiNPs with CaCO_3_-precipitating bacteria induced more significant yield promotion than individual treatment with SiNPs or CaCO_3_-precipitating bacteria (Desoky et al., 2022). In addition, SiNPs can also be applied in conjunction with other NPs in alleviating abiotic stresses. It has been suggested that the combined application of SiNPs and selenium nanoparticles (SeNPs) promoted plant growth and alleviated stress symptoms in strawberry grown under drought stress (Zahedi et al., 2020) and rice grown under Pb exposure (Hussain et al., 2020). In wheat subject to Cd stress, the conjunct application of SiNPs, zinc nanoparticles (ZnNPs), and FeNPs was more effective than other treatments (individual NPs and combination of two NPs) in enhancing grain yield and reducing Cd accumulation (Hussain et al., 2021). In other cases, the synergistic effects of SiNPs and biochar (Alsamadany et al., 2022), indoleacetic acid (IAA) (Sharma et al., 2022), and methyl jasmonate (MeJA) (Moradi et al., 2022) have also been confirmed to be effective in alleviating abiotic stresses such as As stress, Cr stress and salt stress.

## 6 Conclusion and perspective

Overall, this review summarized recent progress of SiNPs application in nano-enabled agriculture, focusing on synthesis, uptake and translocation, and application of SiNPs against various biotic and abiotic stresses. Based on these literatures, it can be concluded that SiNPs application is a cost-effective and multifunctional agronomic approach that is applicable to sustainable agriculture. However, several key issues need further investigation for the more widespread and reasonable usage of SiNPs in agricultural production including: (1) more effective synthesis approach of SiNPs using agricultural wastes; (2) the detailed effects of plant factors (e.g. plant species, plant structures and developmental stages) and SiNPs properties (e.g. size, charge property and specific modification) on the uptake and translocation of SiNPs; (3) the physiological and molecular basis of SiNPs-induced broad-spectrum resistance; (4) the effects and mechanisms of SiNPs modification on their delivery efficiency; (5) the main concerns over potential phytotoxicity induced from the application of SiNPs in agricultural ecosystem.

## Author contributions

GY: Conceptualization, Funding acquisition, Writing – original draft. QH: Writing – original draft. SZ: Writing – original draft. YX: Writing – original draft. YH: Writing – review & editing. MN: Conceptualization, Writing – review & editing. NN: Conceptualization, Writing – review & editing. YL: Conceptualization, Writing – review & editing. ZZ: Conceptualization, Funding acquisition, Writing – review & editing.
